# The untapped potential of oral vitamin E in lichen sclerosus: from immunobiology to real-life clinical experience

**DOI:** 10.3389/fphar.2026.1945302

**Published:** 2026-10-07

**Authors:** Emanuele Scala, Giovanni Luca Scaglione, Lavinia Zozi, Chiara Assorgi, Laura Colonna, Cristina Albanesi, Mauro Picardo, Alessia Paganelli

**Affiliations:** 1 Laboratory of Experimental Immunology, Istituto Dermopatico dell’Immacolata, IDI-IRCCS, Rome, Italy; 2 Bioinformatic Unit, Istituto Dermopatico dell’Immacolata, IDI-IRCCS, Rome, Italy; 3 UniCamillus, Saint Camillus University of Rome, Rome, Italy; 4 Dermatology Unit, Department of Clinical Internal, Anesthesiological and Cardiovascular Science, University of La Sapienza, Rome, Italy; 5 Center for Regenerative Dermatology and Immune-Mediated Skin Diseases, Istituto Dermopatico dell’Immacolata, IDI-IRCCS, Rome, Italy; 6 Department of Life Sciences, Health, and Health Professions, Link Campus University, Rome, Italy

**Keywords:** antioxidant, inflammatory skin diseases, lichen sclerosus, oral supplements, vitamin E

## Abstract

Lichen sclerosus (LS) is a chronic dermatosis characterized by persistent inflammation, progressive fibrosis, and an elevated risk of vulvar squamous cell carcinoma. While ultra-potent topical corticosteroids remain the gold standard of care, the necessity for long-term maintenance therapy raises concerns regarding cumulative toxicity, underscoring the critical need for adjunctive therapies that target underlying pathogenic mechanisms. Vitamin E, traditionally recognized for its antioxidant and membrane-protective properties, has emerged as a promising candidate due to its broad immunomodulatory effects, including its regulatory impact on innate and adaptive immunity and its anti-inflammasome activity. However, the available clinical evidence remains limited to topical formulations, and corresponding efficacy data are scarce. In this Perspective article, we critically analyze the current clinical evidence on oral vitamin E in LS, delineate its immunological mechanisms, and incorporate insights from real-world clinical practice at a tertiary referral center, which should be interpreted as hypothesis-generating given the observational, uncontrolled design. Furthermore, we explore the potential of oral vitamin E formulations, address the pharmacokinetic challenges associated with conventional delivery, and propose future research avenues—such as optimized delivery systems and active metabolite formulations—to fully elucidate the therapeutic role of oral vitamin E in LS management.

## 1 Introduction

Lichen sclerosus (LS) is a chronic, inflammatory dermatosis characterized by progressive sclerosis of the skin and mucosa, with a marked predilection for the anogenital region (Kirtschig, 2016) (Figure 1). Although potentially occurring at any age and in both sexes, vulvar LS represents the most common clinical presentation and typically affects postmenopausal women (McAleer et al., 2026; Rajcigelová et al., 2026). The disease is associated with intense pruritus, burning, pain, dyspareunia, and progressive scarring, leading to anatomical changes affecting sexual function and quality of life (Rouffaneau and Paganelli, 2026). Longstanding LS may also be associated with an increased risk of vulvar squamous cell carcinoma (SCC), highlighting the importance of early diagnosis and long-term disease control (Cooper et al., 2015; Spekreijse et al., 2020).

**FIGURE 1:**
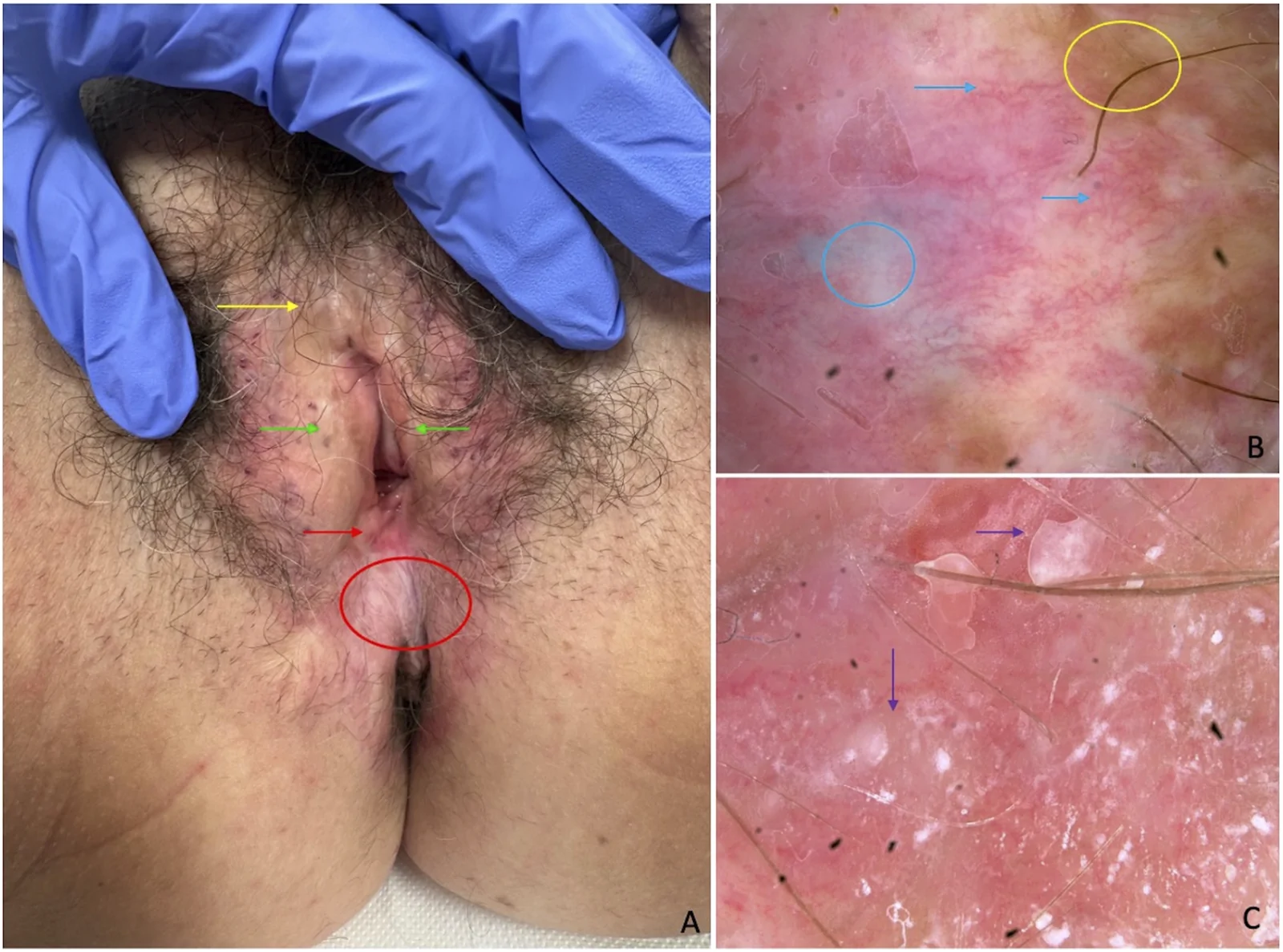
Clinical **(A)** and dermoscopic **(B,C)** images of a patient affected by vulvar lichen sclerosus (LS). Clinically, clitoral phimosis (yellow arrow), fusion of labia minora (green arrows), erosions (red arrow) and whitish atrophic plaques (reed circle) are present **(A)**. Dermoscopy reveals fine telangiectasias (blue arrows), whitish structureless areas (blue circle) and post-inflammatory hyperpigmentation (yellow circle) in the setting of an erythema background **(B)**, as well as whitish hyperkeratotic plaques (purple arrows, C).

Current evidence supports a multifactorial pathogenesis in which genetic susceptibility, autoimmune mechanisms, oxidative stress, and tissue remodeling interact to sustain chronic inflammation and fibrosis (Paganelli et al., 2025). More in detail, local triggers and immune factors contribute to dysregulated extracellular matrix remodeling promoting myofibroblast activation and transforming growth factor-β (TGF-β) release, eventually leading to excessive matrix deposition, and tissue scarring (Pilatz et al., 2013; Sun et al., 2026). Despite significant advances in understanding its pathogenesis, LS remains a chronic relapsing disease requiring lifelong management, often complicated by coexisting autoimmune comorbidities (Paganelli et al., 2024). High-potency topical corticosteroids remain the cornerstone of LS treatment and are highly effective in controlling symptoms and reducing disease activity in most patients (Borghi et al., 2023; Kirtschig et al., 2026). New pathogenetic insights have stimulated interest in therapeutic strategies capable of modulating the biological processes underlying disease progression, including regenerative and immune-directed approaches beyond conventional anti-inflammatory therapy (Paganelli et al., 2023). Nevertheless, complete remission is not universally achieved, relapses are common after treatment discontinuation, and a proportion of patients experience persistent symptoms despite appropriate therapy (Borghi et al., 2018). Long-term maintenance strategies must also be employed to reduce relapse rates once remission has been achieved (Virgili et al., 2013a; Kirtschig et al., 2024). Among these, antioxidant therapies have attracted growing attention because oxidative stress is believed to contribute to both chronic inflammation and tissue damage in LS (Paganelli et al., 2025; Sever et al., 2025). Vitamin E represents one of the best-characterized lipid-soluble antioxidants and has long been recognized for its capacity to protect cellular membranes from oxidative injury. However, increasing experimental evidence indicates that vitamin E exerts biological activities extending well beyond free radical scavenging, including regulation of inflammatory signaling, modulation of immune responses, photoprotection and maintenance of tissue homeostasis (Thiele and Ekanayake-Mudiyanselage, 2007). These pleiotropic properties suggest vitamin E being an attractive candidate for chronic inflammatory disorders such as LS (Ramos-e-Silva et al., 2026), in which immune activation and oxidative stress are central features, and systemic inflammatory markers appear to extend beyond the local anogenital area (Paganelli et al., 2026). Despite this compelling biological rationale, clinical evidence supporting its use remains fragmented.

In this perspective, we revisit the potential role of oral vitamin E in LS by integrating current knowledge from basic immunobiology with the available clinical evidence and complement mechanistic insights with clinical observations.

## 2 Immunobiology of vitamin E

Vitamin E is a family of lipid-soluble compounds collectively known as tocochromanols, whose biological functions extend beyond traditional antioxidant activity to include membrane stabilization, redox regulation, and immune modulation (Lee and Han, 2018; Witt-Wallert et al., 2026). Although α-tocopherol is the predominant, most biologically active, and most extensively studied isoform in humans, emerging evidence indicates that other isoforms—such as γ-tocopherol, δ-tocopherol, tocotrienols, and their metabolites—exert distinct immunoregulatory effects that complement those of α-tocopherol (Szewczyk et al., 2021). Accordingly, α-tocopherol is considered the principal mediator of Vitamin E-dependent protection of both innate and adaptive immunity (Supplementary Figure 1).

Within the innate immune system, α-tocopherol regulates the maturation and function of antigen-presenting cells (APCs), namely, dendritic cells, and modulates macrophage activity in aged mice by downregulating cyclooxygenase-2 (COX-2) expression and reducing prostaglandin E2 (PGE2) synthesis through the inhibition of peroxynitrite production (Beharka et al., 2002). It also shapes the interface between innate and adaptive immunity by controlling intracellular reactive oxygen species (ROS) levels and signaling pathways such as MAPK and NF-κB in dendritic cells, thus limiting excessive antigen presentation and hyperinflammatory responses (Tan et al., 2005; Lee and Han, 2018). Additionally, it supports immune surveillance by increasing natural killer (NK) cell activity (Lee and Han, 2018).

In adaptive immunity, α-tocopherol promotes humoral defense by enhancing B-cell differentiation into plasma cells and increasing antibody production. Concurrently, it boosts T-cell functions by improving early activation signals, promoting cytokine production—particularly IL-2—supporting naïve T-cell proliferation, and maintaining a balanced Th1/Th2 response (Han et al., 2006; Lee and Han, 2018).

Beyond α-tocopherol, other vitamin E isoforms exhibit complementary molecular activities that contribute to inflammation control (Szewczyk et al., 2021). For example, γ-, and δ-tocopherol, as well as tocotrienols inhibit the production of inflammatory lipid mediators, suppress the expression of scavenger receptors such as CD36/FAT involved in foam cell formation, and block activation of the activation of the NLRP3 inflammasome (Wong et al., 2017; Lee and Han, 2018; Szewczyk et al., 2021; Witt-Wallert et al., 2026). These findings underscore the importance of considering the entire vitamin E family when evaluating its immunoregulatory potential.

Metabolism further expands the biological activity of Vitamin E. Tocochromanols are primarily metabolized in the liver into long-chain metabolites, including carboxyethyl-hydroxychromans, which often display greater immunomodulatory and anti-inflammatory activity than their parent compounds (Lee and Han, 2018; Kluge et al., 2019; Szewczyk et al., 2021). Notably, metabolites such as 13′-COOH derived from δ-tocopherol and γ-tocotrienol competitively inhibit COX enzymes by occupying their substrate-binding sites, thereby reducing the synthesis of pro-inflammatory prostaglandins and leukotrienes (Jiang et al., 2011; Pein et al., 2018; Szewczyk et al., 2021). These metabolites also promote a tolerogenic immune phenotypes by modulating MAPK and NFκB signaling, enhancing endotoxin tolerance while limiting pro-inflammatory cytokine production (Jiang et al., 2011; Pein et al., 2018; Szewczyk et al., 2021; Schubert et al., 2022).

In addition, α-tocopherol-derived metabolites, including α-13′-OH and α-13′-COOH, regulate lipid oxidation and endothelial function, thus supporting vascular homeostasis and reducing atherosclerotic processes. They may also inhibit the uptake of oxidized low-density lipoprotein (oxLDL), limiting foam cell formation and vascular inflammation—two key mechanisms underlying metabolic disorders (Wong et al., 2017; Szewczyk et al., 2021).

## 3 Biological rationale in lichen sclerosus

The biological activity of vitamin E positions it as a key immunometabolic and tissue-protective regulator capable of mitigating the aberrant cellular responses that drive LS (Paganelli et al., 2025; Scala and Paganelli, 2026). The pathogenic loop underlying the disease is thought to be initiated in predisposed individuals by “stressed” keratinocytes, which release alarmins, necroptotic mediators, and stress-associated keratins, consequently creating a highly pro-inflammatory microenvironment (van Ee and Garza, 2026). Concurrently, APCs activate T cells by presenting antigens derived from damaged resident skin cells, including fibroblasts, thereby stimulating both humoral and cellular immunity, with a predominant contribution of Th1- and Th17-mediated responses (Beutler et al., 2026; Scala and Paganelli, 2026). In parallel, T-cell-derived interferon-γ (IFN-γ) hyperactivates the JAK/STAT signaling pathway, leading to the upregulation of MHC class I and *de novo* expression of MHC class II molecules on resident skin cells, including keratinocytes, melanocytes and mast cells, and promoting their acquisition of atypical antigen-presenting functions (Albanesi et al., 1998; Beutler et al., 2026). On the other hand, IL-17 exacerbates inflammation, epigenetic dysregulation, stress-response pathways, and fibroblast-mediated extracellular matrix remodeling (Beutler et al., 2026). Consequently, these resident cells further reactivate T cells, reinforcing a self-sustaining pathogenic feedback loop (van Ee and Garza, 2026).

Within this framework, vitamin E, owing to its efficient cellular uptake, could potentially mitigate cellular stress and inflammation within both epidermal and dermal layers. Furthermore, it might help limit excessive antigen presentation by shifting the cytokine profile away from a persistent Th1 polarization and preventing hyperinflammatory priming of skin cells, hence offering a plausible mechanism to disrupt this self-sustaining pathogenic feedback loop at multiple critical junctions. Furthermore, vitamin E, particularly γ-tocotrienol, has been proposed to attenuate the TGF-β/SMAD pathway in smooth muscle cells, thereby limiting the expression of pro-fibrotic genes such as COL1A1 and α-SMA (Zingg, 2015; Fukushima et al., 2017; Qu and Zhao, 2025). Given the central role of TGF-β in the fibrotic stages of LS, targeting this pathway offers potential therapeutic opportunities for refractory or severe cases of LS (Paganelli et al., 2025). However, its effects on non-immune resident skin cells (e.g., keratinocytes and dermal fibroblasts) remain insufficiently characterized. Further experimental studies are necessary to comprehensively elucidate its mechanisms of action and beneficial effects across all skin compartments.

## 4 Evidence-based data from literature search

The role of vitamin E in LS has been investigated over several decades, although the available evidence remains limited and heterogeneous. The earliest report dates back to 1948, when Mackenna and Russell described the oral administration of α-tocopherol (3 mg four times daily) for vulvar LS (Mackenna and Russell, 1948). Despite reported lesion improvement and rapid pruritus relief, this uncontrolled clinical observation did not allow definitive conclusions regarding efficacy. In the late 1980s, interest shifted toward the potential pathogenic role of low systemic vitamin E levels. Two studies evaluated serum vitamin E concentrations in patients with vulvar LS to investigate associations with disease severity, prognosis, or response to etretinate therapy (Romppanen et al., 1985; 1988). Neither demonstrated reduced circulating levels compared with controls or any prognostic relevance, arguing against a systemic vitamin E deficit in vulvar LS. Subsequent research focused primarily on vitamin E as part of combined therapeutic strategies. A Polish case series of 61 patients reported clinical improvement with oral Piascledine® combined with vitamin E in genital and extragenital LS (Brzezińska-Wcisło et al., 2001). Similarly, Borghi and colleagues observed significant improvement in mild-to-moderate vulvar LS using avocado and soybean extracts supplemented with vitamin E (Borghi et al., 2015).

More recently, vitamin E has been explored mainly as a topical adjunctive or supportive treatment. D’Antuono et al. evaluated the use of silk briefs (Dermasilk®) combined with clobetasol propionate and a vitamin E moisturizer, reporting improved symptom control (D’Antuono et al., 2011). In terms of proactive maintenance therapy, a randomized study by Virgili et al. demonstrated that, while mometasone furoate significantly reduced relapse rates, topical vitamin E alone was ineffective in preventing remission, confirming that it cannot replace corticosteroid therapy (Virgili et al., 2013b). However, the same authors suggested that, after stabilization of vulvar LS with topical corticosteroids, long-term use of vitamin E and emollients may be considered as supportive treatment to maintain remission (Virgili et al., 2013a). Other recent trials have investigated formulations containing vitamin E acetate, including combinations with stable ozonides, or vitamin E-based emollients as adjunctive care alongside topical corticosteroids (Currò et al., 2018; Russo et al., 2019). Nevertheless, the therapeutic efficacy of vitamin E—particularly as oral monotherapy—remains unproven, as available studies consistently lack appropriate control groups and monotherapy arms.

## 5 Real-life clinical experience from a tertiary referral center

We conducted an observational single-center study at IDI-IRCCS (Istituto Dermopatico dell'Immacolata–Istituto di Ricovero e Cura a Carattere Scientifico) research hospital (Rome, Italy). The following variables were collected for each patient: sex, age, comorbidities, clinical manifestations, previous or ongoing treatments. The Patient-Administered subscale of the Clinical Scoring System for vulvar LS (PA-CSS) was used for the assessment of disease severity (Günthert et al., 2012). Study procedures were performed in accordance with the principles of the Helsinki Declaration and approved by the local Ethics Committee (EC approval n. 804/1-IDI). Patients with an established clinical and/or histopathological diagnosis of vulvar LS followed up at IDI LS outpatient service, were enrolled in the study. Our goal was to assess the frequency of use as well as the clinical results of oral Vitamin E supplementation among patients with vulvar LS.

In our cohort of 145 women affected by vulvar LS, oral vitamin E supplementation was prescribed in 53 patients (36.6%), reflecting its widespread use as a second-line adjunctive measure within our clinical practice. Administration was guided by three main criteria: (i) failure to achieve complete remission after an induction cycle with topical treatment, (ii) presence of autoimmune systemic comorbidities, and/or (iii) a self-reported antioxidant-poor diet; patients with known allergy or intolerance to vitamin E were excluded. Deeper in detail, all enrolled patients had previously completed the guideline-recommended induction treatment consisting of 3 months of topical clobetasol propionate (Kirtschig et al., 2024), but had had failed to achieve complete remission after this induction phase. At the time of vitamin E supplementation, patients were receiving maintenance therapy only, reflecting a real-life clinical setting. Specifically, 27 patients were on twice-weekly topical corticosteroids, 12 used topical corticosteroids only as needed, and four were on twice-weekly topical calcineurin inhibitors. All patients routinely used emollients as part of their standard supportive care, with 10 subjects receiving emollient therapy alone.

In nearly all cases, oral vitamin E was prescribed in cycles of 3–6 months, administered once or twice yearly, while only a minority of patients reported continuous use for more than 1 year (n = 6). Product-level analysis revealed marked heterogeneity in the formulations prescribed among these 53 patients (Figure 2, panel A). The most frequently used product was an avocado and soybean extract containing phytosterols (45 mg) and tocopherol (36 mg DL-α-tocopheryl acetate), accounting for 28 patients (52.8%), followed by tocopherol (RRR-α-tocopherol) 400 IU alone in 12 patients (22.6%) and a tocotrienol (40 mg)/tocopherol (10 mg) formulation combined with vitamin F in 11 patients (20.8%). Two additional formulations—Vitamin E (α-tocopheryl acetate) 70 mg combined with vitamin A 30,000 IU, and a tocotrienol/tocopherol (30 mg D-α-tocopherol) preparation combined with Bifidobacterium brevis BR03 and *Lactobacillus rhamnosus* LR05—were each used in a single patient. This variability underscores the current absence of a standardized oral vitamin E regimen for LS in real-world practice, with clinicians and patients drawing from a heterogeneous pool of commercially available antioxidant and nutraceutical formulations rather than a single validated product or dosage.

**FIGURE 2:**
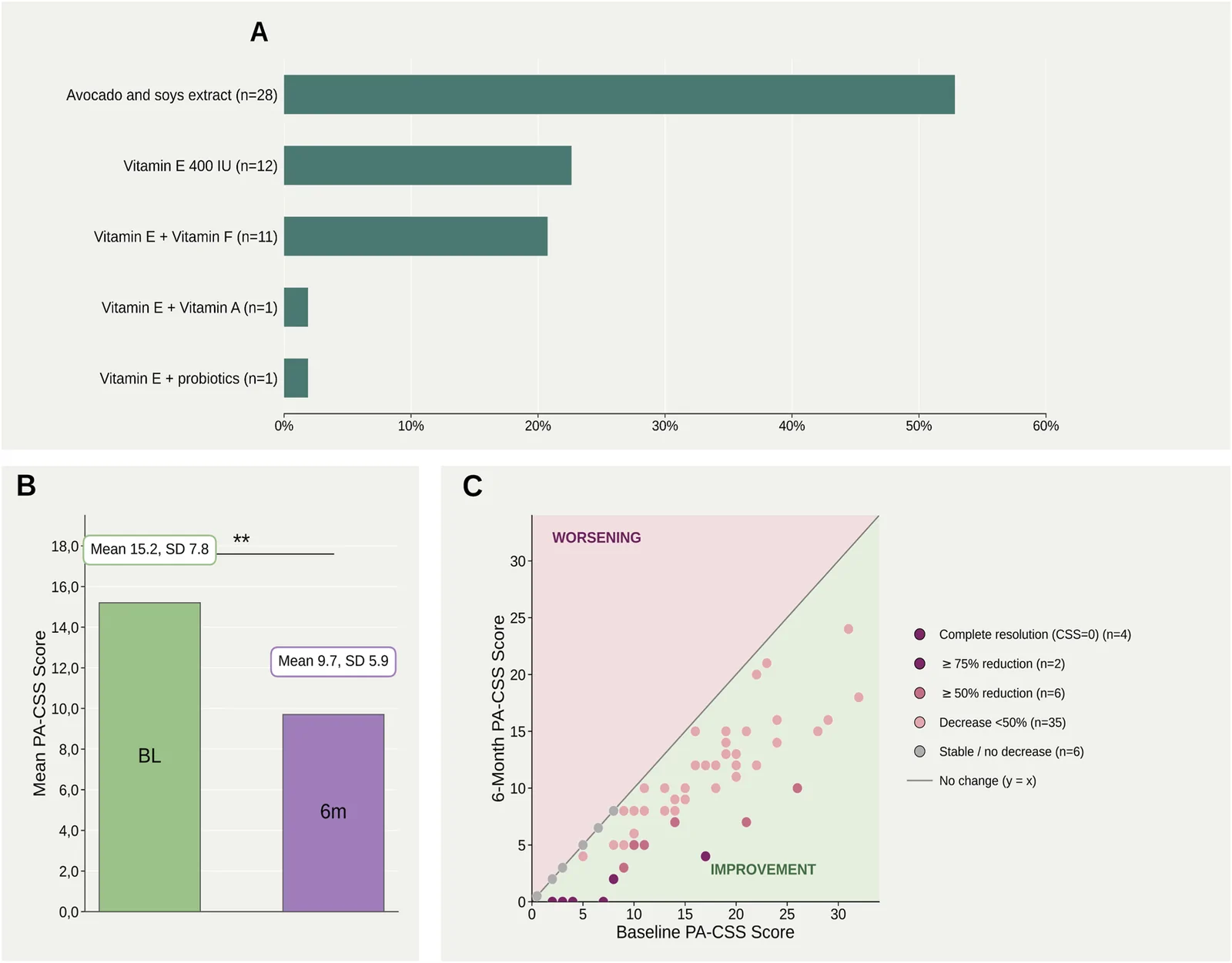
**(A)** Absolute count and relative percentages of vitamin E based supplements among the 53 patients reporting an oral vitamin E product use. **(B)** Histogram representation of mean Patient-Administered CSS change between baseline (BL, green) and the 6-month follow up (6m, purple). **(C)** Individual baseline Vs. 6-month CSS by response category (n = 53).

Among our cohort, the PA-CSS showed a significant reduction from baseline (BL) to the 6-month follow-up (Figure 2, panel B). BL PA-CSS values ranged widely (mean 15.2, SD 7.8; median 15, IQR 9–20), reflecting a heterogeneous population in terms of disease activity at treatment initiation. After 6 months, mean PA-CSS decreased to 9.7 (SD 5.9; median 9, IQR 5–14), corresponding to a mean absolute reduction of 5.5 points (SD 4.0) and a mean relative reduction of 37.3% from baseline. This improvement was statistically significant (paired t-test, p < 0.0001; Wilcoxon signed-rank test, p < 0.0001). At the individual level (Figure 2, panel C), 47 of 53 patients (88.7%) showed a decrease in PA-CSS, 6 (11.3%) remained stable, and none worsened. A clinically meaningful response, defined as a ≥50% reduction in PA-CSS from BL, was observed in 12 patients (22.6%), while six patients (11.3%) achieved a ≥75% reduction. Complete symptomatic resolution (PA-CSS = 0) was documented in four patients (7.5%). Taken together, these findings suggest that oral vitamin E, when used as an adjunctive measure, may be associated with a consistent improvement in patient-reported symptoms of vulvar LS.

Despite these encouraging results, we have to acknowledge several limitations of our study. First, different vitamin E-containing formulations included in this real-world cohort also contained additional bioactive compounds, such as phytosterols, probiotics, vitamin A, or other components, which may have contributed to the observed clinical effects. Additionally, vitamin E was not randomly assigned but preferentially prescribed to patients with incomplete remission after induction therapy, autoimmune comorbidities, or a self-reported antioxidant-poor diet, introducing a selection bias that may limit generalizability to the broader LS population. Furthermore, all patients were concurrently receiving maintenance therapy (topical corticosteroids, calcineurin inhibitors, or emollients) at the time of vitamin E supplementation, meaning that vitamin E was never evaluated as a true monotherapy. Second, it should also be noted that in this real-life setting we relied exclusively on the patient-administered symptom score (pruritus, burning, soreness, and dyspareunia), and not on the classical physician-administered CSS assessing objective signs (erosions, hyperkeratosis, fissures, agglutination, stenosis, and atrophy), due to the short follow-up period and the lack of a single dedicated examiner across visits, which would have limited the reliability of an objective architectural assessment. Consequently, a score of 0 reflects complete symptomatic remission as perceived by the patient, rather than full morphological normalization of vulvar architecture, which is known to be largely irreversible in advanced SL. Moreover, the 6-month follow-up period is too short to establish the durability of benefit, long-term safety, or the impact of vitamin E on scarring and relapse rates. No biomarkers of oxidative stress, inflammatory cytokines, histological changes, or tissue vitamin E levels were measured, so the immunological mechanisms proposed above remain speculative in relation to our clinical observations. Finally, because patients were aware of receiving a supplement with an expected therapeutic benefit and outcomes relied entirely on patient-reported measures, expectation and placebo effects may have inflated the apparent clinical response. Lastly, owning to the observational real-world design and the absence of a control group, our findings do not establish a causal effect of vitamin E supplementation but rather suggest a potential association with standard maintenance regimens that warrants confirmation in controlled prospective studies.

## 6 Discussion

Taken together, evidence gathered across historical data and our own tertiary-center experience portrays oral vitamin E as a biologically plausible but clinically underdeveloped adjunct in the management of vulvar LS (a possible preliminary algorithm for vitamin E supplementation is shown in Supplementary Figure 2).

The rationale for systemic immunomodulation in LS has gained support from increasing evidence that the disease extends beyond a purely localized cutaneous disorder. The identification of circulating autoantibodies directed against extracellular matrix protein 1 (ECM1) provided the first strong indication of a systemic autoimmune component (Oyama et al., 2003). Subsequent studies confirmed the presence of both humoral and cell-mediated abnormalities, reinforcing the notion of LS as a localized manifestation of systemic immune disfunction (Gambichler et al., 2013). More recently, alterations in peripheral inflammatory markers, including increased neutrophil-to-lymphocyte ratios (NLR), have provided additional evidence of systemic immune activation in LS (Paganelli et al., 2026). In parallel, the frequent association of LS with autoimmune comorbidities further supports a complex immunological background and suggests shared inflammatory pathways extending beyond the vulvar mucosa (Corazza et al., 2021; Paganelli et al., 2024). These observations strengthen the biological rationale for interventions capable of modulating inflammation and oxidative stress at a systemic level.

The immunological rationale for Vitamin E use in LS is convincing: tocochromanols and their long-chain metabolites target the immune response dampening NF-κB–and MAPK–driven inflammatory signaling, limiting NLRP3 inflammasome activation, restraining pro-inflammatory eicosanoid synthesis, and supporting a more tolerogenic immune phenotype (Tan et al., 2005; Lee and Han, 2018). All these pathways are directly relevant to persistent immune activation and progressive dermal fibrosis that define LS. Consistent with this rationale, our real-world cohort showed a significant reduction in patient-reported PA-CSS scores at 6 months among patients receiving oral vitamin E as an adjunctive measure. However, this improvement was, on average, partial rather than curative, and occurred against a backdrop of markedly heterogeneous formulations, doses, and treatment schedules. Such findings mirror the fragmentation already evident in the published literature, where vitamin E has almost never been tested as a standardized oral monotherapy against an appropriate control arm.

This gap between mechanistic promise and modest clinical benefit is unlikely to reflect a lack of biological activity alone. Despite a compelling immunological rationale, clinical translation faces substantial pharmacokinetic challenges. Oral vitamin E bioavailability is inherently limited by variable intestinal absorption, competition among isoforms for hepatic α-tocopherol transfer protein, and rapid hepatic catabolism (Kluge et al., 2019; Szewczyk et al., 2021). These factors constrain the dose reaching peripheral inflamed tissue and may explain why even biologically active isoforms fail to sustain therapeutic concentrations at the vulvar dermal-epidermal interface.

This pharmacokinetic bottleneck may account for the discrepancy between the receptor- and pathway-level evidence supporting vitamin E’s immunomodulatory potential and the comparatively modest responses observed clinically, including in our own cohort, where prescribing patterns were largely empirical and rarely sustained beyond a 3–6-month cycle. Addressing this bottleneck will likely require moving beyond conventional oral supplementation: optimized formulations, targeted strategies, and advanced delivery platforms directing vitamin E and its bioactive metabolites toward inflamed tissues may be necessary to translate the immunological rationale into a reproducible clinical effect. Until such delivery-optimized approaches are tested in adequately controlled trials, oral vitamin E should be regarded as a rational but pharmacokinetically constrained adjunct complementing first-line topical corticosteroid therapy in the long-term management of LS and, potentially, other chronic inflammatory dermatoses sharing similar immunopathological features. In this context, our study should be regarded as a pilot, hypothesis-generating investigation, intended to provide preliminary real-world evidence and to stimulate future randomized studies evaluating standardized vitamin E formulations capable of isolating the specific contribution of individual vitamin E isoforms.

## Data Availability

The raw data supporting the conclusions of this article will be made available by the authors, without undue reservation.
